# Temperament and Character Domains of Personality and Depression 2012

**DOI:** 10.1155/2012/946725

**Published:** 2012-11-11

**Authors:** Toshinori Kitamura, C. Robert Cloninger, Andrea Fossati, Jörg Richter

**Affiliations:** ^1^Kitamura Institute of Mental Health Tokyo, 101 Minato 8-5-13, Tokyo 107-0052, Japan; ^2^Department of Psychiatry, Washington University in St. Louis, 660 South Euclid Avenue, St. Louis, MO 63130, USA; ^3^Faculty of Psychology, Vita-Salute San Raffaele University, 20132 Milan, Italy; ^4^Centre for Child and Adolescent Mental Health, Ulleval University Hospital, 0424 Oslo, Norway

*Depression Research and Treatment* has issued a second compendium of papers focused on temperament, character, and depression. The psychobiology theory of personality proposed by Cloninger is a currently prevailing theory of personality that has been extensively investigated in the context of many different types of mental and personality disorders. This special issue reports up-to-date research findings on the psychobiology theory and depression from different countries.

The current issue consists of six reports. Miettunen and colleagues in Finland present findings from a longitudinal birth cohort study (*N* = 4941). Participants with depression at 31 years of followup had higher rates of harm avoidance (HA) than participants without any psychiatric disorders. Participants without any psychiatric history were followed for another 12 years. Those who subsequently developed depression had high HA in 1997. The authors hypothesize that high HA is a potential indicator for subsequent depression. This study only used temperament scales and thus no information was available on the association between character and depression.

Students in senior high schools (*N* = 1234) who were invited to participate in an internet-based intervention program for depression were studied by Christian and colleagues in Norway. High HA and low self-directedness (SD) emerged as strong predictors of adolescent depression. Interestingly, use of the internet intervention program was associated with low reward dependence (RD) in addition to depression severity.

Garcia and colleagues studied an adolescent population (*N* = 304) in Sweden. Based on positive affect (PA) and negative affect (NA) scores derived from the Positive Affect and Negative Affect Schedule, the participants were categorized into four groups: self-fulfilling (high PA and low NA), high affective (high PA and high NA), low affective (low PA and low NA), and self-destructive (low PA and high NA). The self-fulfilling group was characterized by higher persistence (PS), SD, and cooperativeness (CO) than the three other groups. The self-destructive group was characterized by high HA and RD. The authors claim character maturity (expressed as high SD and CO) is important for psychological well-being.

In Japan, Lu and colleagues followed graduate students (*N* = 184) on two occasions separated by a five-month interval. In a structural regression model, they posited that trait anxiety and depression constructs were linked to high HA and low SD. Although trait anxiety and depression scores were moderately correlated with each other, these two constructs showed different associations with Temperament and Character Inventory (TCI) subscale scores. Thus, trait depression was linked to high self-transcendence (ST) whereas trait anxiety was linked to low RD, PS, and CO. The authors claim that character maturity is linked to trait rather than state aspects of depression and anxiety.

Directly exposed survivors of the Oklahoma City bombing randomly selected from a bombing survivor registry (*N* = 151) were examined by North and colleagues in the USA. Posttraumatic stress disorder (PTSD) after the bombing was associated with low SD and CO together with high ST and HA. Postdisaster major depression (MD) was more prevalent among those with PTSD than those without it, but low SD and CO could not be predicted by post-disaster MD. The authors emphasized the importance of developing and validating measures of resilience.

A unique measure of temperament, the Temperament Evaluation of Memphis, Pisa, Paris, and San Diego (TEMPS), was used by Tei-Tominaga and colleagues to study job stress among employees in one Japanese company (*N* = 728). Depression was predicted by high levels of cyclothymic and anxious temperament traits even after controlling for the effects of work-related stressors such as demanding work conditions and overcommitment.

These articles all indicate the importance of temperament and character traits in the development of depression among a variety of populations across different countries. Despite some differences between the studies, a common theme may be that low SD and high HA are predictors of depression either directly or being mediated by third variables.

*Toshinori Kitamura* *Toshinori Kitamura* *C. Robert Cloninger* *C. Robert Cloninger* *Andrea Fossati* *Andrea Fossati* *Jörg Richter* *Jörg Richter*

